# Syndromic ciliopathy: a taiwanese single-center study

**DOI:** 10.1186/s12920-024-01880-0

**Published:** 2024-04-26

**Authors:** Yu-Wen Pan, Tsung-Ying Ou, Yen-Yin Chou, Pao-Lin Kuo, Hui-Pin Hsiao, Pao-Chin Chiu, Ju-Li Lin, Fu-Sung Lo, Chung-Hsing Wang, Peng-Chieh Chen, Meng-Che Tsai

**Affiliations:** 1 Department of Pediatrics, College of Medicine, National Cheng Kung University Hospital, National Cheng Kung University, No. 138, Shengli Rd., North Dist, Tainan, 70403 Taiwan, Republic of China; 2 Department of Genomic Medicine, College of Medicine, National Cheng Kung University Hospital, National Cheng Kung University, No. 138, Shengli Rd., North Dist, Tainan, 70403 Taiwan, Republic of China; 3 Department of Gynecology and Obstetrics, College of Medicine, National Cheng Kung University Hospital, National Cheng Kung University, No. 138, Shengli Rd., North Dist, Tainan, 70403 Taiwan, Republic of China; 4 Institute of Clinical Medicine, College of Medicine, National Cheng Kung University, No. 138, Shengli Rd., North Dist, Tainan, 70403 Taiwan, Republic of China; 5 Center of Clinical Medicine, College of Medicine, National Cheng Kung University Hospital, National Cheng Kung University, No. 138, Shengli Rd., North Dist, Tainan, 70403 Taiwan, Republic of China; 6 Department of Pediatrics, Dalin Tzu Chi Hospital, Buddhist Tzu Chi Medical Foundation, No. 2, Minsheng Rd., Dalin Township, Chiayi County, Chiayi, 62247 Taiwan, Republic of China; 7 Department of Pediatrics, Kaohsiung Medical University Chung Ho Memorial Hospital, No. 100, Ziyou 1st Rd., Sanmin Dist, Kaohsiung, 80756 Taiwan, Republic of China; 8 Department of Pediatrics, Kaohsiung Veterans General Hospital, No. 386, Dazhong 1st Rd., Zuoying Dist, Kaohsiung, 813414 Taiwan, Republic of China; 9 Department of Pediatrics, Chang Gung Children’s Hospital, No. 5, Fuxing St., Guishan Dist, Taoyuan, 333423 Taiwan, Republic of China; 10 Division of Genetics and Metabolism, Children’s Hospital of China Medical University, No. 2, Yude Rd., North Dist, Taichung, 404327 Taiwan, Republic of China; 11 School of Medicine, China Medical University, No. 91, Xueshi Rd., North Dist, Taichung, 404328 Taiwan, Republic of China; 12 Department of Obstetrics and Gynecology, E-Da Hospital, No. 1, Yida Rd., Yanchao Dist, Kaohsiung, 824005 Taiwan, Republic of China

**Keywords:** Ciliopathy, Bardet-biedl syndrome, Alström syndrome, Oral-facial-digital syndrome, Joubert syndrome, Whole exome sequencing

## Abstract

**Background:**

Syndromic ciliopathies are a group of congenital disorders characterized by broad clinical and genetic overlap, including obesity, visual problems, skeletal anomalies, mental retardation, and renal diseases. The hallmark of the pathophysiology among these disorders is defective ciliary functions or formation. Many different genes have been implicated in the pathogenesis of these diseases, but some patients still remain unclear about their genotypes.

**Methods:**

The aim of this study was to identify the genetic causes in patients with syndromic ciliopathy. Patients suspected of or meeting clinical diagnostic criteria for any type of syndromic ciliopathy were recruited at a single diagnostic medical center in Southern Taiwan. Whole exome sequencing (WES) was employed to identify their genotypes and elucidate the mutation spectrum in Taiwanese patients with syndromic ciliopathy. Clinical information was collected at the time of patient enrollment.

**Results:**

A total of 14 cases were molecularly diagnosed with syndromic ciliopathy. Among these cases, 10 had Bardet-Biedl syndrome (BBS), comprising eight BBS2 patients and two BBS7 patients. Additionally, two cases were diagnosed with Alström syndrome, one with Oral-facial-digital syndrome type 14, and another with Joubert syndrome type 10. A total of 4 novel variants were identified. A recurrent splice site mutation, *BBS2*: c.534 + 1G > T, was present in all eight BBS2 patients, suggesting a founder effect. One BBS2 patient with homozygous c.534 + 1G > T mutations carried a third ciliopathic allele, *TTC21B*: c.264_267dupTAGA, a nonsense mutation resulting in a premature stop codon and protein truncation.

**Conclusions:**

Whole exome sequencing (WES) assists in identifying molecular pathogenic variants in ciliopathic patients, as well as the genetic hotspot mutations in specific populations. It should be considered as the first-line genetic testing for heterogeneous disorders characterized by the involvement of multiple genes and diverse clinical manifestations.

**Supplementary Information:**

The online version contains supplementary material available at 10.1186/s12920-024-01880-0.

## Introduction

Primary cilia are crucial intracellular structures composed of microtubules. Structurally, the ciliary axoneme, which is comprised of nine doublet microtubules, is surrounded by a lipid bilayer membrane and assembles from a basal body consisting of two centrioles from the centrosome [1].

There are two types of cilia: motile and immotile. Unlike motile cilia, primarily found in certain tissues where they transport extracellular fluid over the epithelial surface, immotile cilia exist in almost all cell types and serve different functions. They are responsible for numerous critical cellular actions related to tissue development and physiological functions [2, 3]. Utilizing microtubules as an intracellular scaffold, ciliary motor proteins can transport cargo proteins and organelles along the ciliary axoneme. Immotile cilia also play a role in sensing extracellular signals, which is crucial for tissue morphogenesis and homeostasis [4]. Accordingly, defects in these ciliary functions or formations, collectively referred to as ciliopathies, can result in a constellation of overlapping clinical features. These features may include obesity, retinal degeneration, renal disease, bone abnormalities, and cerebral anomalies, which can manifest themselves across different disease entities. Some ciliopathic patients may only exhibit defective renal development, such as polycystic kidney diseases, while others may exhibit syndromic disorders like Bardet-Biedl syndrome (BBS), Alström syndrome (ALMS), Joubert syndrome (JS), Meckel-Gruber syndrome (MKS), or Oral-Facial-Digital syndrome (OFDS). Despite the rarity of syndromic ciliopathy, the multisystemic pathology has imposed significant physiological and psychological burdens on affected families. Additionally, the genetic heterogeneity of syndromic ciliopathy has made molecular diagnosis challenging when a single-gene approach, such as Sanger sequencing, is adopted. To date, more than 200 genes have been reported to be associated with these phenotypically overlapping diseases, and the number of causative genes continues to grow [5, 6]. In this study, we present the genotypes and phenotypes of a group of Taiwanese patients who were molecularly confirmed to have syndromic ciliopathy through whole exome sequencing.

## Materials and methods

### Patient cohort

Subjects were referred for genetic testing by their treating physicians from local facility or tertiary center across all regions of Taiwan to a tertiary medical center in Southern Taiwan if they had received a tentative clinical diagnosis of a syndromic ciliopathy, such as BBS, ALMS, JS, MKS, OFDS, or any other rarer type. As syndromic ciliopathies comprise a clinically heterogeneous disorder encompassing neurodevelopmental, skeletal, renal ciliopathy, and syndromic obesity [2], we classified the clinical manifestations into major and minor criteria. The major criteria include mental retardation, renal structural anomalies or insufficiency, skeletal anomalies such as polydactyly, overweight/obesity, hypogonadism/ambiguous genitalia, and ocular disease or visual impairment. The minor criteria consist of impaired glucose tolerance, hepatic steatosis, hearing impairment, and congenital heart disease or heart dysfunction. Among the referred cases, individuals who met at least three major criteria or two major criteria plus two minor criteria were selected for whole exome sequencing (WES). Individuals with known genetic causes for other syndromic disorders were excluded. Informed consent of either themselves or their proxy guardians were obtained. Clinical information, including phenotype records, was collected. The entire procedure was approved by the Institutional Review Board of the National Cheng Kung University Hospital (A-BR-109-045 and B-BR-104-063).

### Whole exome sequencing and variants analysis

Genomic DNA of the proband was extracted from a peripheral blood sample. The exome library was generated using SureSelect^QXT^ All human exon V6 (Agilent), which covers around 60 Mb of the exonic regions and 20,000 genes. Paired-end sequencing was performed on the Illumina NextSeq 500 sequencer. Sequencing reads were aligned to human genome reference Hg19 using Novoalign [7]. Single nucleotide variants and small insertions and deletions were called with Genome Analysis Toolkit 3.4 (GATK) [8]. Variants were annotated with ANNOVAR [9] and novel variants were filtered against dbSNP [10], 1000 Genome SNP [11] and Genome Aggregation Database [12]. Variants were then sorted according to the Combined Annotation Dependent Depletion (CADD) score [13]. The functional effects of these amino acid substitutions were predicted using in silico analysis tools, including PolyPhen2 [14], PROVEAN [15], SIFT [16], MutationTaster [17], and varSEAK [18]. Sanger sequencing was performed for the probands, and when available, their parents, to validate the findings and confirm de novo variants for dominant inheritance or in trans variants for recessive inheritance. Furthermore, we conducted a variant-based pairwise kinship analysis, calculating kinship coefficients using VCFTOOLS-relatedness2 function, to determine the relatedness between each pair of individuals. An estimated kingship coefficient ranges greater than 0.354, from 0.177 to 0.354, from 0.0884 to 0.177, and from 0.0442 to 0.0884 corresponds to duplicate/Monozygotic twin, 1st-, 2nd-, and 3rd degree relationships, respectively [19].

## Results

**Fig. 1:**
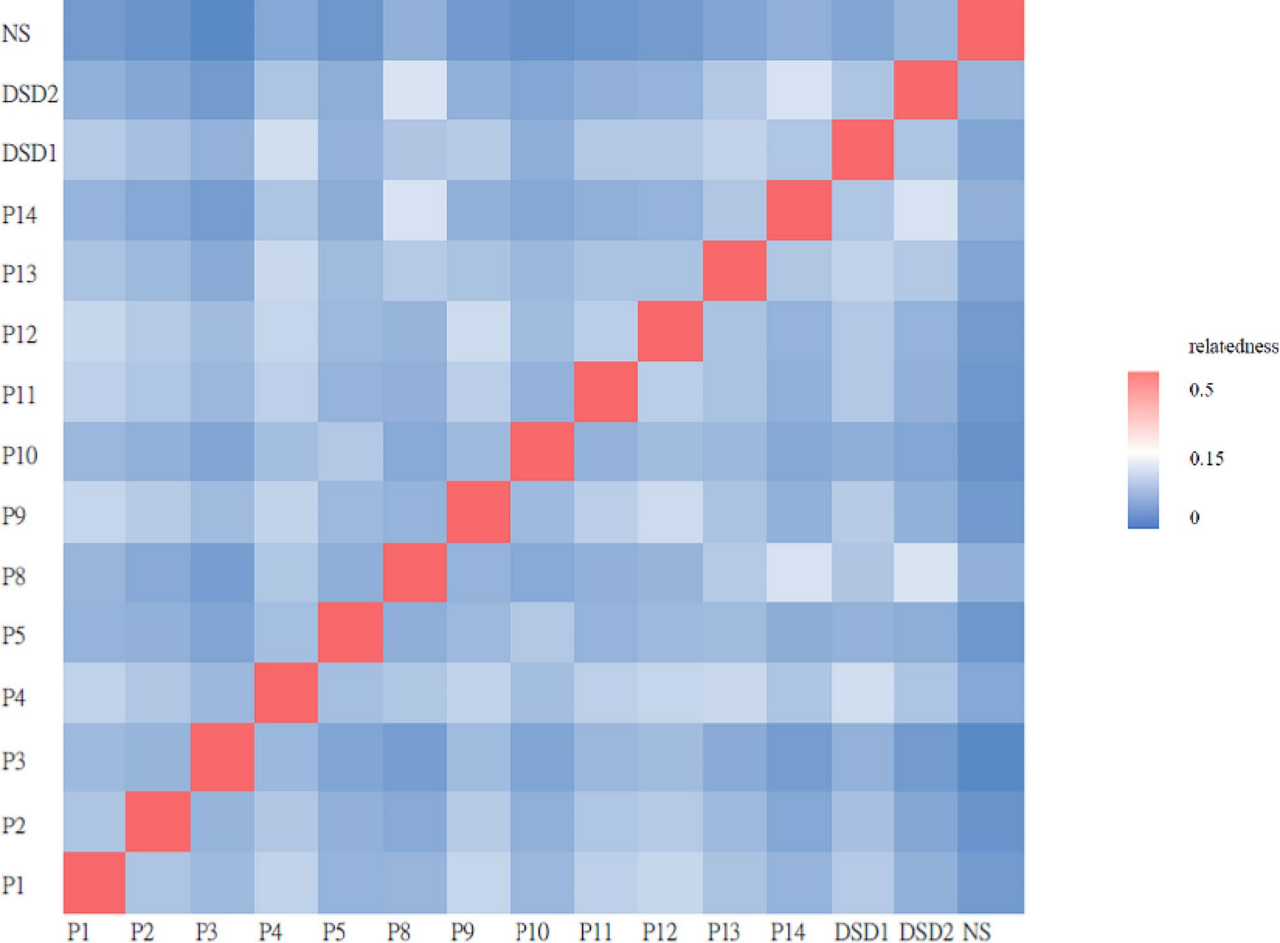
Pairwise kinship coefficients were calculated using VCFTOOLS relatedness2 function to determine the relatedness between each pair of cases in our cohort, which included two Taiwanese patients with a disorder of sex development (DSD) and one Caucasian with Noonan syndrome (NS) serving as controls. P6 and P7 were excluded from the analysis since they are siblings. The kinship values ranged from the lowest at 0.043826 to the highest at 0.127509. The Caucasian with Noonan syndrome was the most unrelated individual to the other cases, while the Taiwanese individuals, regardless of having ciliopathy or DSD, exhibited similar kinship values, reflecting the genetic background of the Taiwanese population

**Fig. 2:**
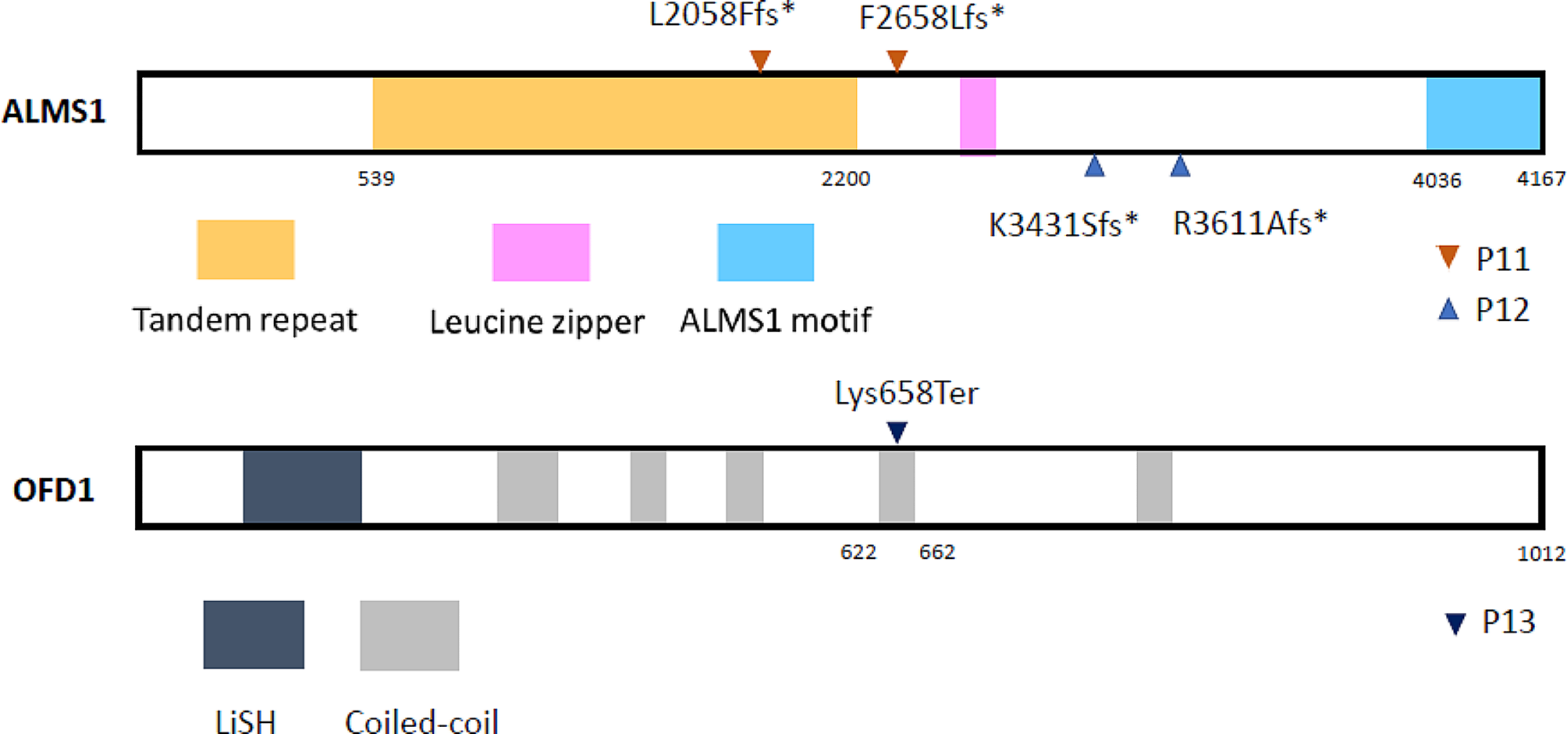
(**A**) ALMS1 protein and the truncated mutations of P10 and P11 on *ALMS1* gene (**B**) OFD1 protein and the truncated mutation of P13 on *OFD1* gene

**Fig. 3:**
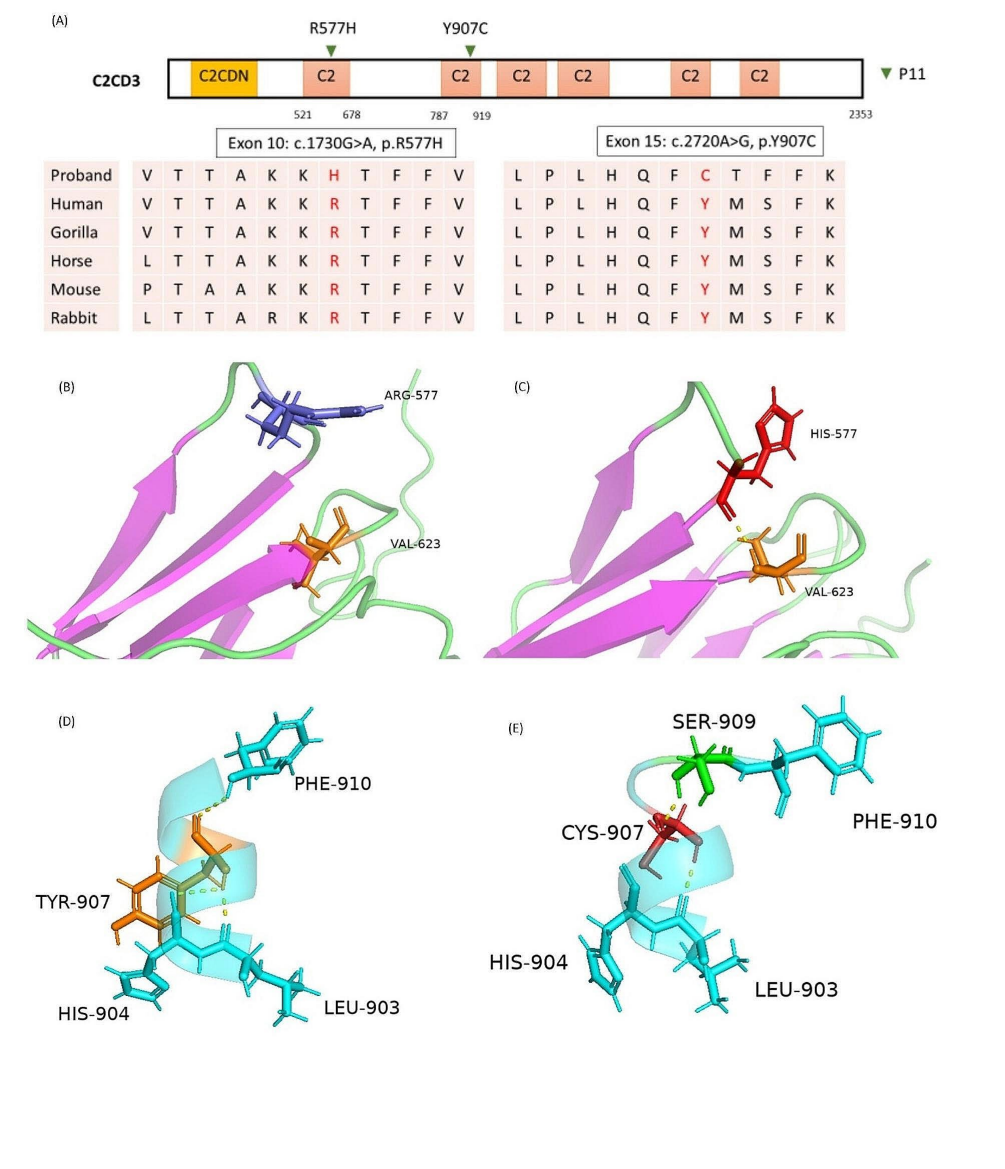
C2CD3 (C2 calcium-dependent domain containing 3) protein has six canonical PKC-C2 domains and a C2CD3N–C2 domain at the N-terminus [25]. The graph showed the conserved amino acid residues at the 577 and 907 positions on C2CD3 protein (**A**). The iterative threading assembly refinement (I-TASSER) server was used to predict the three-dimensional structure of wild type (**B** and **D**) and mutant (**C** and **E**) C2CD3 protein. The yellow dashed line indicates the hydrogen bond. (**C**) The H577 residue is predicted to form a new hydrogen bond with V623 residue, which slightly changed the β-sheet structure. (**E**) The C907 residue is predicted to cause hydrogen bond loss with H904 and F910 and form a new hydrogen bond with the S909 residue, affecting the proper protein folding in the α‐helix

From 2015 to 2022, we consecutively enrolled 14 cases, belonging to 13 unrelated families, who were clinically suspected of syndromic ciliopathy. All of them were born into non-consanguineous families. The kingship coefficients between individuals were plotted in Heatmap as Fig. 1. We identified causal homozygous or compound heterozygous mutations in the following genes: *BBS2* in eight patients, *BBS7* in two patients, *ALMS1* in two patients, and *C2CD3* in one patient. Additionally, one patient had a hemizygous mutation in the *OFD1* gene. Among the variants we identified, four were novel, including one frameshift mutation in *BBS7*, one frameshift mutation in *ALMS1*, one missense mutation in *C2CD3*, and the other nonsense mutation in *OFD1*. Three of the four novel mutations were found in a compound heterozygous state, and the other was a hemizygous mutation on the X chromosome. The genotypic information is listed in Table 1, supplementary Table 1, Figs. 2 and 3, and the patients’ characteristics are detailed in Tables 2 and 3, and Table 4.

**Table 1:** Genotypes of the 14 patients with syndromic ciliopathy

Gene	Patient	Variants	Location	Types	Amino acid substitutions	Allele frequency in east asian ^a^	Prediction	American college of medical genetics and genomics (ACMG) prediction [20]
*BBS2* (NM_031885.5)	P1	c.534 + 1G > T	IVS4	Splice donor	NA	1.4*10^− 3^	varSEAK: exon skipping	Pathogenic PVS1, PM3, PP3, PP4, PP5
		c.1814 C > G	Exon15	Nonsense	p.Ser605Ter	5.4*10^− 5^	MutationTaster: disease causing	Pathogenic PVS1, PM2, PM3, PP3, PP4, PP5
	P2	c.534 + 1G > T	IVS4	Splice donor	NA			
		c.563del	Exon5	Frameshift	p.Ile188Thrfs*13	1.1*10^− 4^	MutationTaster: disease causing	Pathogenic PVS1, PM2, PM3, PP3, PP4, PP5
	P3	c.534 + 1G > T	IVS4	Splice donor	NA			
		c.534 + 1G > T	IVS4	Splice donor	NA			
	P4	c.534 + 1G > T	IVS4	Splice donor	NA			
		c.534 + 1G > T	IVS4	Splice donor	NA			
		***TTC21B***: c.264_267dupTAGA (NM_024753.5)	Exon4	Nonsense	p.E90Ter	8.6*10^− 4^	MutationTaster: disease causing	Pathogenic PVS1, PM2, PP3, PP5
	P5	c.534 + 1G > T	IVS4	Splice donor	NA			
		c.563del	Exon5	Frameshift	p.Ile188ThrfsTer13			
	P6	c.534 + 1G > T	IVS4	Splice donor	NA			
		c.534 + 1G > T	IVS4	Splice donor	NA			
	P7	c.534 + 1G > T	IVS4	Splice donor	NA			
		c.534 + 1G > T	IVS4	Splice donor	NA			
	P8	c.534 + 1G > T	IVS4	Splice donor	NA			
		c.534 + 1G > T	IVS4	Splice donor	NA			
*BBS7* (NM_176824.3)	P9	c.728G > A	Exon8	Missense	p.Cys243Tyr	5.5*10^− 4^	Polyphen2: probably damaging SIFT: damaging PROVEAN: deleterious MutationTaster: disease causing	Likely pathogenic PM2, PM3, PP2, PP3, PP4, PP5
		c.1685_1686del	Exon16	Frameshift	p.Glu562GlyfsTer4	Novel	MutationTaster: disease causing	Pathogenic PVS1, PM2, PP3, PP4
	P10	c.849 + 1G > C	IVS8	Splice donor	NA	2.7*10^− 4^	varSEAK: exon skipping	Pathogenic PVS1, PM2, PP3, PP4
		c.728G > A	Exon8	Missense	p.Cys243Tyr			
*ALMS1* (NM_015120.4)	P11	c.6169_6170dupAT	Exon8	Frameshift	p.Leu2058PhefsTer17	1.6*10^− 4^	MutationTaster: disease causing	Pathogenic PVS1, PM2, PM3, PP3, PP4
		c.7972_7978del	Exon10	Frameshift	p.Phe2658LeufsTer25	Novel	MutationTaster: disease causing	Pathogenic PVS1, PM2, PM3, PP3, PP4
	P12	c.10831_10832del	Exon 16	Frameshift	p.Arg3611AlafsTer6	3.5*10^− 4^	MutationTaster: disease causing	Pathogenic PVS1, PM2, PM3, PP3, PP4
		c.10290_10291del	Exon 15	Frameshift	p.Lys3431SerfsTer10	0 ^b^	MutationTaster: disease causing	Pathogenic PVS1, PM2, PM3, PP3, PP4
*OFD1* (NM_003611.3)	P13	c.1972 A > T	Exon 16	Nonsense	p.Lys658Ter	Novel	MutationTaster: disease causing	Pathogenic PVS1, PM2, PP3, PP4
*C2CD3* (NM_015531.6)	P14	c.2720 A > G	Exon15	Missense	p.Tyr907Cys	Novel	Polyphen2: probably damaging SIFT: damaging PROVEAN: deleterious MutationTaster: disease causing	Likely pathogenic PM1, PM2, PP3, PP4
		c.1730G > A	Exon10	Missense	p.Arg577His	0	Polyphen2: probably damaging SIFT: tolerated PROVEAN: neutral MutationTaster: disease causing	Likely pathogenic PM1, PM2, PP3, PP4

^a^ The allele frequency is referenced by gnomAD Exomes, Version 2.1.1

^b^ It was a known allele and reported by us in 2018 but the allele frequency is 0 in population database

**Table 2:** Phenotypes of the patients with bardet biedl syndrome (BBS)

Patients	Sex	Age	Diagnosis	Primary features						Secondary features
				Overweight/ Obesity	Visual impairment	Polydactyly	Renal disease	Mental retardation	Hypogonadism/ genital anomalies	
P1	M	30	BBS type 2	Y	Y	Y	Y	Y	N	
P2	F	19	BBS type 2	Y	Y	Y	N	Y	Y	Hepatic steatosis
P3	M	8	BBS type 2	Y	Y	Y	N	Y	Y	
P4	M	7	BBS type 2	Y	Y	Y	N	Y	Y	
P5	F	14	BBS type 2	Y	Y	Y	Y	Y	Y	Right atrial isomerism Asplenia
P6	M	14	BBS type 2	Y	Y	N	Y	Y	N	Brachydactyly
P7	F	13	BBS type 2	Y	Y	Y	N	Y	N	
P8	F	21	BBS type 2	Y	N	Y	Y	N	Y	
P9	F	19	BBS type 7	Y	Y	Y	Y	Y	Y	
P10	M	14	BBS type 7	N	Y	Y	Y	Y	Y	Strabismus/Cataracts

**Table 3:** Phenotypes of the patients with Alström syndrome (ALMS)

Patients	Sex	Age	Diagnosis	Major feature	Minor features						
				Visual impairment	Overweight/ Obesity	(History of) cardiomyopathy	Hearing loss	Renal failure	Hepatic dysfunction	Hypogonadism/ genital anomalies	Short stature
P11	M	8	ALMS	Y	Y	Y	N	N	N	Y	N
P12	M	16	ALMS	Y	Y	Y	Y	Y	Y	Y	Y

**Table 4:** Phenotypes of the patients with Joubert syndrome XX and Oral-facial-digital syndrome (OFDS) XIV

Patients	Sex	Age	Diagnosis	Clinical manifestations
P13	M	3	Joubert syndrome type XX	Bilateral optic disc coloboma Renal cyst Hypotonia Developmental delay Molar tooth sign on brain MRI Polymicrogyria Failure to thrive
P14	M	15	OFDS type XIV	Dysmorphism Microcephaly Trigonocephaly Ambiguous genitalia Mental retardation

### Bardet-biedl syndrome (BBS)

A total of 10 patients were molecularly diagnosed with Bardet-Biedl syndrome. Among them, eight patients had bi-allelic mutations in the *BBS2* gene, and all of them harbored at least one IVS4 donor splice site mutation, c.534 + 1G > T, predicted to cause a splicing effect on the mRNA transcript by varSEAK (Table 1). Three of the eight patients carried compound heterozygous mutations, while the others had homozygous mutations of this variant. None of the eight BBS2 patients had additional variants in other BBS-causative genes. However, one patient (P4) had an additional protein-truncating mutation, c.264_267dupTAGA, in *TTC21B*, a gene also involved in ciliary function (Table 1, supplementary Fig. 1). Two patients carried bi-allelic compound heterozygous mutations in the *BBS7* gene. One of the identified *BBS7* variants (c.1685_1686del, p.Glu562GlyfsTer4) in P7 was a novel frameshift mutation predicted to be pathogenic by MutationTaster (Table 1). It is located in exon 16 and is predicted to result in a premature stop codon and protein truncation. The two patients diagnosed with BBS7 carried neither additional pathogenic variants in other BBS-causative genes nor variants in other ciliopathic genes. All of the patients clinically met the diagnostic criteria for BBS, presenting with at least four primary features or three primary features plus two secondary features [21]. The most prevalent presentations included obesity (90%), ocular diseases (90%), polydactyly (90%), and mental retardation (90%) (Table 2).

### Alström syndrome (AS)

Patient 11 and Patient 12 presented with clinical manifestations resembling those of BBS (Table 3) [22]. Whole exome sequencing (WES) identified bi-allelic compound heterozygous mutations in the *ALMS1* gene in both patients. Patient 12 was previously reported by us in 2018 [23]. He carried a rare frameshift mutation in exon 15 (c.10290_10291del) and another rare frameshift mutation in exon 16 (c.10831_10832del). WES for Patient 11 identified a rare frameshift mutation in exon 8 (c.6169_6170dupAT) inherited from his father and a novel frameshift mutation in exon 10 (c.7972_7978del) inherited from his mother. The compound heterozygous mutations identified in both patients are predicted to cause premature termination of translation of the ALMS1 protein (Fig. 2).

### Joubert syndrome (JS) type 10

Whole exome sequencing (WES) for P13 revealed a hemizygous mutation in the *OFD1* gene, c.1972 A > T, which was inherited from his mother (Table 1, supplementary Fig. 1). Based on characteristic neurodevelopmental abnormalities and the distinctive molar tooth sign (MTS) observed on brain magnetic resonance imaging (MRI), he was diagnosed with Joubert syndrome, type 10. This identified variant was a novel nonsense mutation predicted to be pathogenic by MutationTaster (Table 1). It affected the amino acid residue at position 658 and was predicted to cause premature protein truncation of the OFD1 protein (Fig. 2).

### Oral-facial-digital (OFD) syndrome type 14

Patient 14 exhibited more prominent dysmorphic facial features and skeletal abnormalities compared to the other patients in our cohort. He did not exhibit obesity and visual problems, which were the most prevalent clinical manifestations in patients with BBS and ALMS. Instead, he had microcephaly, trigonocephaly, and dysmorphic features such as a beaked nose, helmet-like face, micrognathia, small mouth and lips, and ambiguous genitalia as major presentations. Whole exome sequencing (WES) for patient 14 identified compound heterozygous mutations in the *C2CD3* gene. Both mutations were missense mutations and were predicted to be pathogenic in silico (Table 1). One of these variants, c.2720 A > G (p.Tyr907Cys), was novel and affected the conserved amino acid residue 907 in the 2nd canonical C2 functional domain of C2CD3 (Fig. 3). The other rare variant, not previously observed in East Asians, also affected the conserved amino acid at position 577 in the 1st canonical C2 domain (Fig. 3). The two mutations are predicted to induce a three-dimensional structural change in the C2CD3 protein using the iterative threading assembly refinement (I-TASSER) server [24] (Fig. 3).

## Discussion

In this report, we describe a total of 14 Taiwanese patients who were molecularly diagnosed with a specific type of ciliopathy through WES. These patients exhibited overlapping clinical features, including obesity, visual problems, skeletal anomalies, mental retardation, and renal diseases. In our cohort, we identified a total of four novel variants, including *BBS7*: c.1685_1686del, *ALMS1*: c.7972_7978del, *OFD1*: c.1972 A > T, and *C2CD3*: c.2720 A > G. These findings contribute to the expansion of genotypic information for patients with syndromic ciliopathy.

Among the patients we diagnosed, the majority were identified as BBS patients, which is likely due to BBS serving as a model disease for syndromic ciliopathy. We identified a novel mutation: *BBS7* c.1685_1686del, p.Glu562GlyfsTer4. This frameshift mutation is predicted to result in premature protein truncation and has been classified as pathogenic by multiple predictive in silico software tools. The *BBS7* gene encodes one of eight proteins that form the BBSome complex, which functions to sort specific membrane proteins to the primary cilia [26]. P9 carries compound heterozygous mutations with *BBS7*: c.728G > A, p.Cys243Tyr (C243Y), and *BBS7*: c.1685_1686del, p.Glu562GlyfsTer4 (E562fs). C243Y, though without functional assay, has been previously reported in a compound heterozygous or homozygous state in BBS7 patients [27, 28], while E562fs is a truncated mutation, classically resulting in a loss-of-function mutation. This combination of mutations provides a clear and unequivocal genetic etiology for P9.

Within the subset of eight patients diagnosed with BBS2, the splice donor site mutation c.534 + 1G > T, predicted to cause exon skipping, was present in all eight cases (8/8). Four of these patients were homozygous for this mutation, while the remaining individuals were compound heterozygous. c.534 + 1G > T in the *BBS2* gene was recently identified in a Chinese BBS cohort. It was observed in two out of the five patients diagnosed with BBS2, existing in a compound heterozygous state. However, the researchers couldn’t determine if this allele was a common occurrence in the Chinese population due to the limited number of cases included in the study [28]. In contrast, our study revealed that 100% (8/8) of the BBS2 patients in our cohort, originating from non-consanguineous families and diverse regions across Taiwan, carried this allele. Given that this allele is currently exclusive to East Asian populations, we are more confident that c.534 + 1G > T is the hotspot mutation for the Taiwanese population. These findings also suggest a potential founder effect for this pathogenic variant within the Taiwanese population. Although the kinship coefficient between individuals with BBS2 showed relatedness to some degree (Kinship coefficients range from the lowest at 0.061596 to the highest at 0.126118), these values are similar to that conducted between pairs of BBS2 and other diseases, such as disorders of sex development (DSD) (Kinship coefficients range from the lowest at 0.0602572 to the highest at 0.127509). Additionally, among 93,000 Taiwanese individuals in Taiwan Biobank, 26,693 (28.8%) were found to have at least one related individual within the third degree or closer [29]. These findings reflect the genetic background of the Taiwanese population.

There has been a wide discussion about the oligogenic or tri-allelic inheritance of BBS patients previously due to the wide inter- and intra-familial variability of clinical manifestations [30–32]. However, no conclusion has been made yet. Besides, several studies provided evidence of a second-site modifier effect on BBS phenotypes [33–35]. In our study, none of our cases had an additional BBS-associated mutation that supported tri-allelism. All molecularly confirmed BBS patients conformed to the autosomal-recessive pattern of Mendelian inheritance rules. Nevertheless, a third pathogenic allele in *TCC21B* was identified in one patient (P4) with homozygous *BBS2* mutations. *TTC21B* is recognized not only as the causal gene for Nephronophthisis (NPHP) and Jeune asphyxiating thoracic dystrophy (JATD) but also as a potential modifying factor across the spectrum of ciliopathies. It is suggested to be a common contributor to the total mutational load in a broad range of disease entities in ciliopathy [36]. Furthermore, a few ciliopathic cases presenting with atypical or exaggerated phenotypes were reported to carry an additional *TTC21B* pathogenic allele [37, 38]. These findings led us to further investigating the modifying effect of the *TTC21B* allele in the phenotype of P4. After reviewing the clinical manifestations of the P4 case in our study, he did not exhibit atypical or exaggerated symptoms/signs at the time of recruitment when he was only seven years old. However, it should be emphasized that the majority of ciliopathies may present their clinical symptoms/signs progressively. Symptoms/signs that are not evident at a young age may emerge over time and require further monitoring. In conclusion, the contributing effect of the *TTC21B* allele in our case is inconclusive, despite the fact that it is predicted to be pathogenic. Further studies with a larger sample size and segregation data or functional assay are needed to investigate the effects of TTC21B on BBS phenotypes.

Alström syndrome (ALMS) exhibits overlapping clinical features similar to those of BBS, making it clinically challenging to differentiate between ALMS and BBS without genetic testing. The causative gene of ALMS, *ALMS1*, encodes a large protein with 23 exons and more than 4000 residues, primarily participating in the formation and maintenance of cilia. ALMS1 lacks known catalytic domains but incorporates several sequence features of unknown function, including an ALMS1 motif at the C-terminus. The function of this motif has not been fully elucidated, but it may be essential for centrosomal localization [39, 40]. To date, the majority of the identified ALMS1 variants are nonsense or frameshift mutations, resulting in truncated proteins that lack the ALMS1 motif [40]. Notably, exons 8, 10, and 16 are mutational hotspots within the *ALMS1* gene [41]. In our study, three out of the four identified variants in ALMS patients, including a novel mutation, c.7972_7978del, were located within these mutational hotspot exons, and all translated proteins are predicted to be truncated, lacking the ALMS1 motif.

Oral-facial-digital syndrome (OFDS) is a complex syndromic disorder with at least 18 different subtypes identified to date. Due to the heterogeneous and overlapping clinical manifestations, differentiating between these subtypes of OFD syndrome clinically without genetic testing remains challenging [42]. A few cases of OFDS were reported in Taiwan in the past but were diagnosed clinically without genetic testing at that time. Our case (P14), carrying bi-allelic mutations of *C2CD3*, is, to the best of our knowledge, the first OFDS XIV case and the first genetically confirmed OFDS case in Taiwan to date. In addition to the oral, facial, and digital malformations, OFD syndrome type XIV is characterized by the presence of severe microcephaly, trigonocephaly, severe intellectual disability, and micro-penis, all of which were present in our patient. *C2CD3* encodes a centriolar protein that regulates centriole elongation and distal appendage assembly at the base of cilia, both of which are required for primary cilia biogenesis. It contains multiple evolutionarily conserved C2 domains, including a C2CD3N-C2 domain at the N-terminus and six canonical PKC-C2 domains, which are believed to be involved in protein-protein interactions [25, 43, 44]. Most of the reported mutations causing OFD XIV in C2CD3 are located within these C2 domains [45]. The novel variant identified in P14: C2CD3, c.2720 A > G, p.Tyr907Cys, is also located at one of the C2 domain in C2CD3 protein.

Like C2CD3, the OFD1 protein is also involved in primary cilia biogenesis [46, 47]. The OFD1 protein contains a Lis homology (LisH) domain in its N-terminal region and several coiled-coil (CC) domains located C-terminal to the LisH motif. These CC domains have been demonstrated to be critical for centrosomal targeting [48]. Most reported OFD1 mutations lead to truncated proteins lacking the CC domains [48, 49]. WES for P13 identified a novel truncated mutation within the CC domain. He was clinically diagnosed with Joubert syndrome based on the typical triad of clinical manifestations including hypotonia, developmental delays, and the presence of a molar tooth sign on brain MRI before the molecular diagnosis was made.

Our study has limitations. We did not conduct additional functional assays to validate the pathogenicity of the novel variants identified, including those found in *BBS7*, *ALMS*, *OFD1*, and *C2CD3*. Additionally, the modifier effect of TTC21B on the BBS2 phenotype was not evaluated through a functional assay, as most of the patients’ blood samples were not available. Despite predicting pathogenicity in silico for these variants, a functional assay provides experimental evidence regarding the impact of these variants on gene function or protein activity, which is essential for drawing conclusive conclusions.

## Conclusion

In conclusion, next-generation sequencing should be considered as the first-line genetic testing for heterogeneous disorders in which multiple genes are involved and diverse clinical manifestations are presented. It greatly aids in the molecular diagnosis of syndromic ciliopathy. Furthermore, it helps identify genetic hotspots in specific populations and novel variants in known causative genes. It also allows for the investigation of complex inheritance patterns, such as tri-allelism. Future research necessitates a larger cohort to better elucidate genotype-phenotype correlations. Additionally, conducting functional assays that compare mutant variants with wild-type counterparts is imperative to validate the pathogenicity of identified variants.

### Electronic supplementary material

Below is the link to the electronic supplementary material.

Supplementary Material 1

Supplementary Material 2

Supplementary Material 3

## Data Availability

The datasets generated and analyzed during the current study are available in the ClinVar repository, [Accession number: SCV004042735, SCV004042815, SCV004042839, SCV004046853, SCV004045996, SCV004045997.1, SCV004046645.2, SCV004046846, SCV004046847, SCV004046848, SCV004046849.2, SCV004046850, SCV004046851.1, SCV004046852.2].
