# Supplementary figures and images for: Syndromic ciliopathy: a taiwanese single-center study
Source: BMC Med Genomics. 2024 Apr 26;17:106. doi: 10.1186/s12920-024-01880-0 (PMC11046915; doi:10.1186/s12920-024-01880-0)

P5

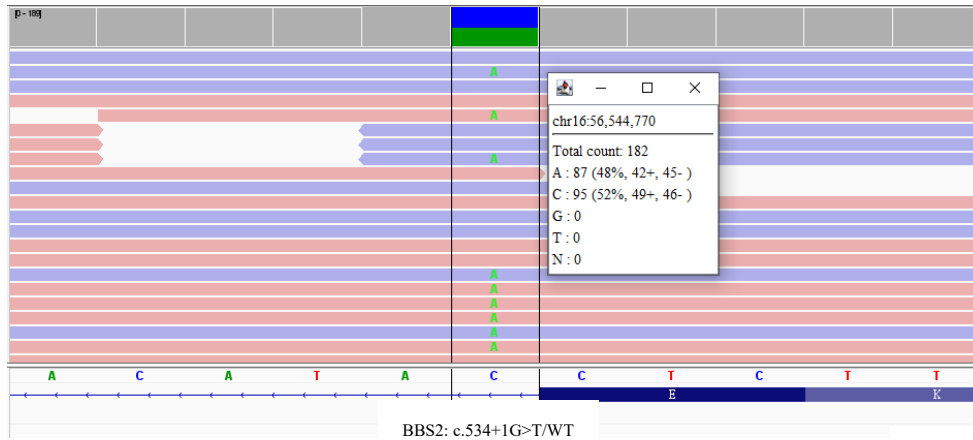

P10

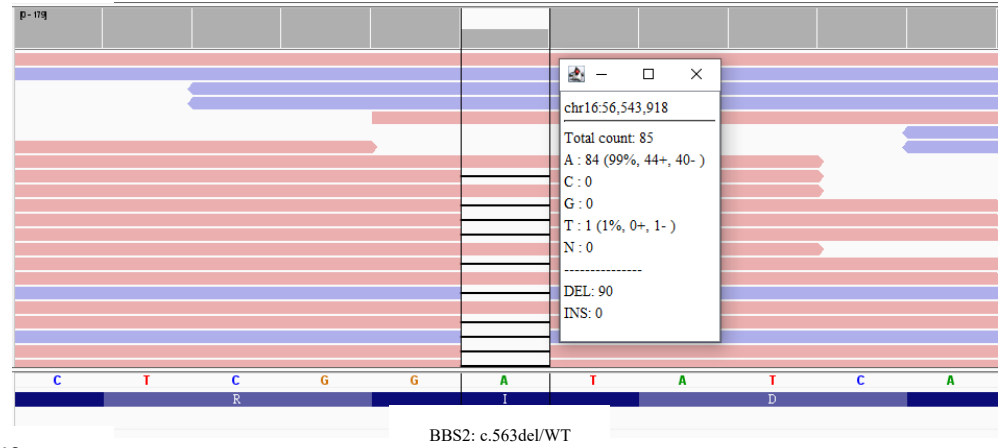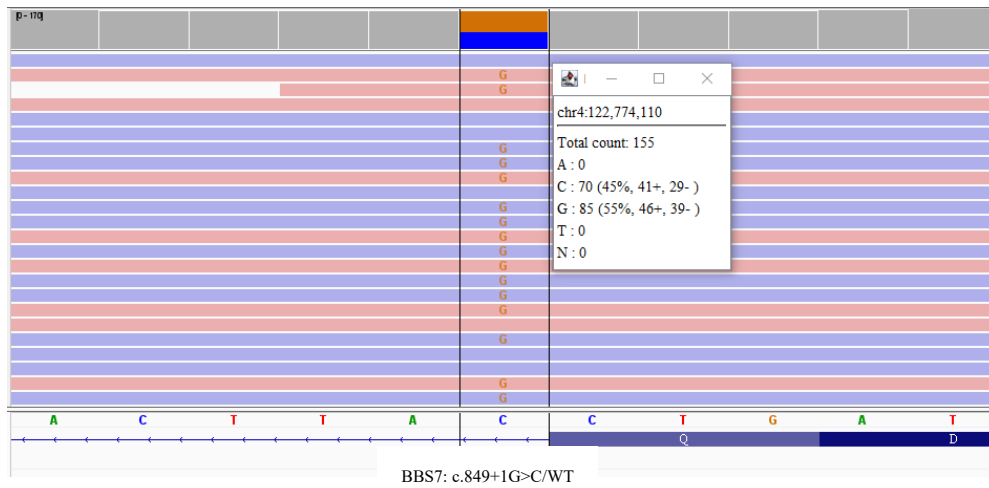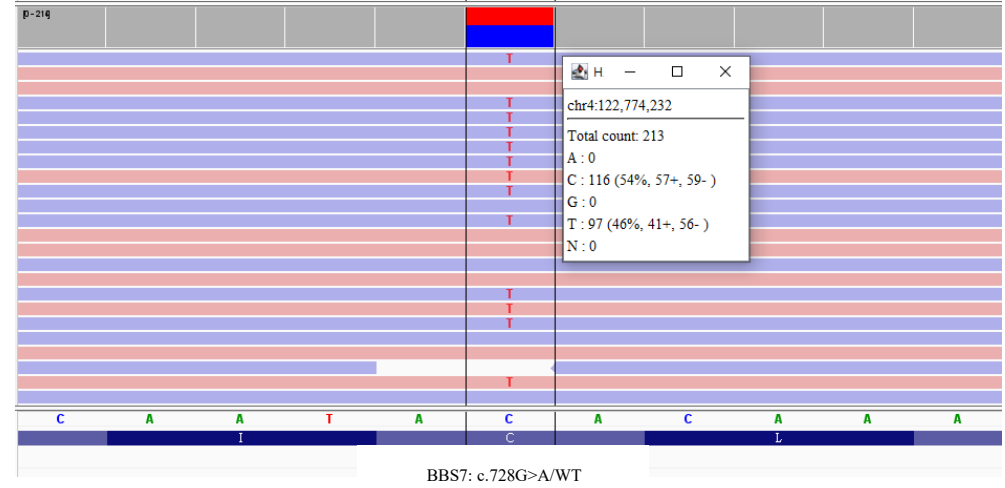

Supplementary Figure 2. Mutational sequences of P5 and P10

Supplement: Supplementary file 1 — Supplementary Material 1 [file 12920_2024_1880_MOESM1_ESM.pdf]
