# Supplementary material for: Syndromic ciliopathy: a taiwanese single-center study
Source: BMC Med Genomics. 2024 Apr 26;17:106. doi: 10.1186/s12920-024-01880-0 (PMC11046915; doi:10.1186/s12920-024-01880-0)
Supplement: Supplementary file 2 — Supplementary Material 2 [file 12920_2024_1880_MOESM2_ESM.pdf]

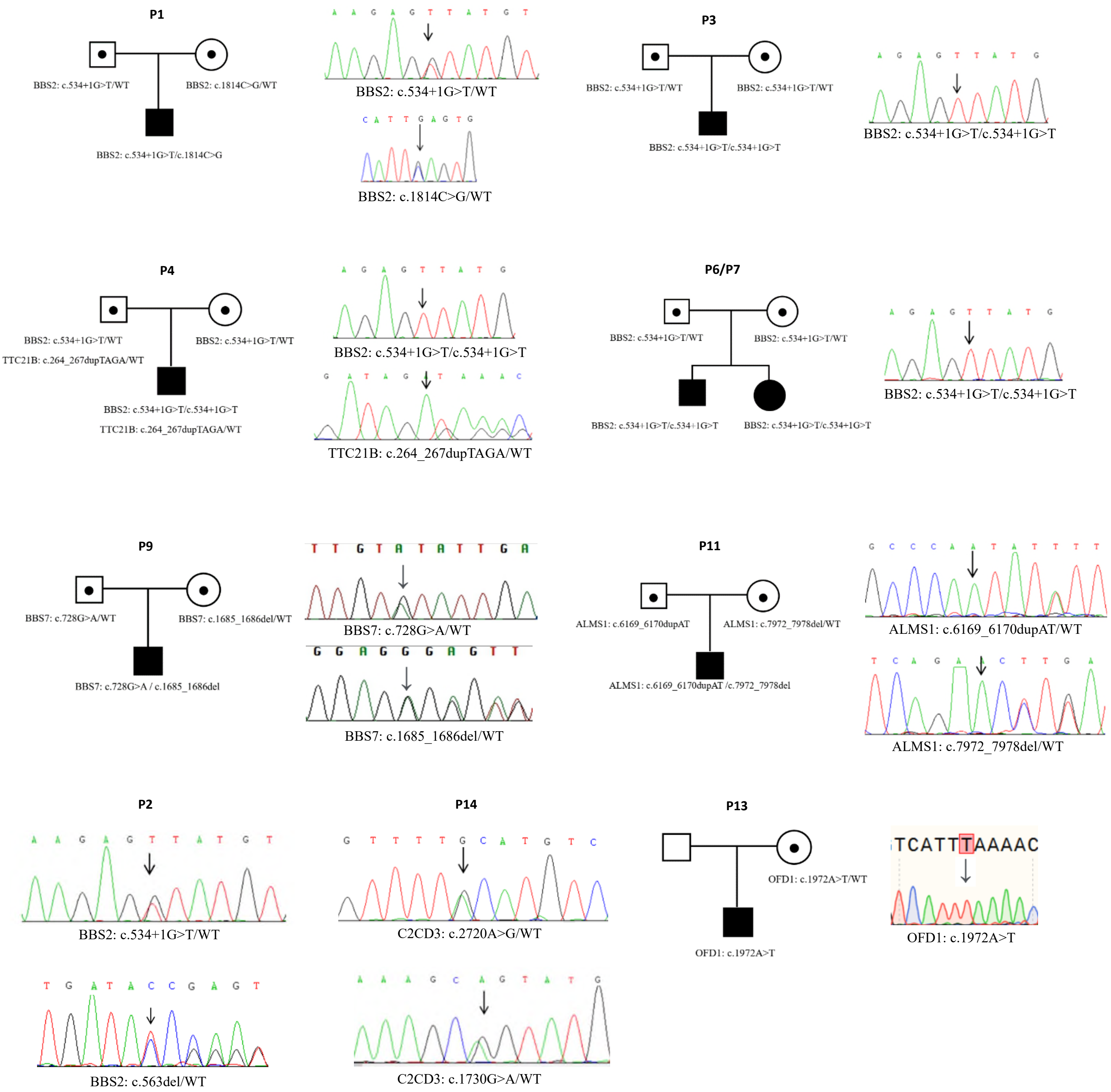

Supplementary Figure 1. Pedigrees and the mutational sequences of patients with syndromic ciliopathy (P1, P2, P3, P4, P6/P7, P9, P11, P13, and P14)
