# Supplementary material for: Syndromic ciliopathy: a taiwanese single-center study
Source: BMC Med Genomics. 2024 Apr 26;17:106. doi: 10.1186/s12920-024-01880-0 (PMC11046915; doi:10.1186/s12920-024-01880-0)
Supplement: Supplementary file 3 — Supplementary Material 3 [file 12920_2024_1880_MOESM3_ESM.pdf]

Supplementary table 1. The Human Genome Variation Society (HGVS) nomenclature of genetic variants in our cohort

Gene	HGVS nomenclature
**BBS2**	NC_000016.9:g.56544770C>A; NM_031885.5:c.534+1G>T; NP_114091.4:p.?
**BBS2**	NC_000016.9:g.56530975G>C; NM_031885.5:c.1814C>G; NP_114091.4:p.(Ser605Ter)
**BBS2**	NC_000016.9:g.56543918del; NM_031885.5:c.563del; NP_114091.4:p.(Ile188ThrfsTer13)
**TTC21B**	NC_000002.11:g.166802199_166802202dup; NM_024753.5:c.264_267dup; NP_079029.3:p.(Glu90Ter)
**BBS7**	NC_000004.11:g.122774232C>T; NM_176824.3:c.728G>A; NP_789794.1:p.(Cys243Tyr)
**BBS7**	NC_000004.11:g.122749877_122749878del; NM_176824.3:c.1685_1686del; NP_789794.1:p.(Glu562GlyfsTer4)
**BBS7**	NC_000004.11:g.122774110C>G; NM_176824.3:c.849+1G>C; NP_789794.1:p.?
**ALMS1**	NC_000002.11:g.73679820_73679821dup; NM_015120.4:c.6169_6170dup; NP_055935.4:p.(Leu2058PhefsTer17)
**ALMS1**	NC_000002.11:g.73717055_73717061del; NM_015120.4:c.7972_7978del; NP_055935.4:p.(Phe2658LeufsTer25)
**ALMS1**	NC_000002.11:g.73799832_73799833del; NM_015120.4: c.10831_10832del; NP_055935.4:p.(Arg3611AlafsTer6)
**ALMS1**	NC_000002.11:g.73786172_73786173del; NM_015120.4:c.10290_10291del; NP_055935.4:p.(Lys3431SerfsTer10)
**OFD1**	NC_000023.10:g.13778551A>T; NM_003611.3:c.1972A>T; NP_003602.1:p.(Lys658Ter)
**C2CD3**	NC_000011.9:g.73811582T>C; NM_015531.6:c.2720A>G; NP_056346.3:p.(Tyr907Cys)
**C2CD3**	NC_000011.9:g.73825429C>T; NM_015531.6:c.1730G>A; NP_056346.3:p.(Arg577His)
